# Reformation Capability of Short-Range Order and Their Medium-Range Connections Regulates Deformability of Bulk Metallic Glasses

**DOI:** 10.1038/srep12177

**Published:** 2015-07-16

**Authors:** Congling Li, Yujie Wei, Xinghua Shi

**Affiliations:** 1 LNM, Institute of Mechanics, Chinese Academy of Sciences, Beijing 100190; 2 School of Civil Engineering, Luoyang Institute of Science and Technology, Luoyang, Henan Province 471023.

## Abstract

Metallic glasses (MGs) typically have high yield strength while low ductility, and the latter is commonly considered as the Achilles’ heel of MGs. Elucidate the mechanism for such low ductility becomes the research focus of this field. With molecular level simulations, we show the degree of short-range order (SRO) of atomic structure for brittle Fe-based glass decreases dramatically during the stretch, while mild change occurs in ductile Zr-based glass. The reformation capability for SRO and their medium-range connections is found to be the primary characteristics to differentiate the deformability between the two metallic glasses. We suspect that, in addition to the strength of networks formed by SRO structure, the reformation capability to reform SRO networks also plays the key role in regulating the ductility in metallic glasses. Our study provides important insights into the understanding about the mechanisms accounting for ductility or brittleness of bulk metallic glasses.

Bulk metallic glasses (BMGs) have captured significant interests due to their unique properties such as high stiffness, high toughness and superior corrosion resistance^1^,^2^,^3^,^4^,^5^,^6^,^7^. However the low ductility of BMGs below their glass transition temperature *T*_g_ restricts their wide applications. Searching for BMGs with both high strength and high ductility thus becomes the current focus of research. So far, the properties associated with deformability in BMGs have been explored from both macroscopic and atomistic scales. For the former, it is found that the plasticity of BMGs is closely linked to the Poisson’s ratio of the material: BMGs show significant toughness when the Poisson’s ratio exceeds a critical value of 0.31–0.32^8^,^9^, with greater Poisson’s ratio leading to more diffusive shear bands^10^. Further studies suggest specimen aspect ratio^11^, free volume^12^,^13^,^14^, loading rate^11^,^14^ and energy dissipative mechanisms^12^ maybe have influence on the plasticity of MGs. On the atomic level, Zhang *et al.*^15^ explained the plasticity observed in Zr–Cu(Ni)–Al MGs based on internal atomistic structures from simulations. A strategy was then proposed to design BMGs compositions with the desired local order which is considered as the structural basis for BMGs^16^,^17^. How the local order structures improve the ductility, however, remains elusive. Cheng *et al.*^18^ then resolved the atomic structure of Cu_46_Zr_47_Al_7_ through molecular dynamics (MD) simulation and found the small percentage of Al leads to dramatically increased population of Cu/Al centered full icosahedra (FI) as well as their spatial connectivity and medium-range order (MRO). Kumar *et al.*^19^ found that substituting a few percent of Al for Zr in Cu_46_Zr_54_ MGs leads to remarkable increase in strength and ductility. Follow this line, Lee *et al.*^20^ investigated the evolution of MRO during applied loading and found the interpenetrating connection of icosahedra (ICOI) network is resistant to stress-induced shear transformation. The disruption of ICOI network would result in obvious strain localization and lower plasticity. This finding alone, however, cannot explain the experimental observation of ductility enhancement^19^. It is desirable to explore the influence of SRO on the ductility of MGs thoroughly, especially to see how the formation of shear transformation zone (STZ) is mediated by SRO. In this work we conduct comparative MD simulations on Zr-based and Fe-based MGs, and the former is typically considered as ductile material while the latter is brittle^21^. By uniaxially stretching Zr-based and Fe-based nanowires and bulk samples with cracks, we observe that although the SRO network resists the formation of STZ, the reformation of SRO and their medium-range connections can effectively block the development of shear localization when it initiates, and hence enhances the ductility of MGs.

## Results

### Ductility of MG nanowires

**Figure 1:**
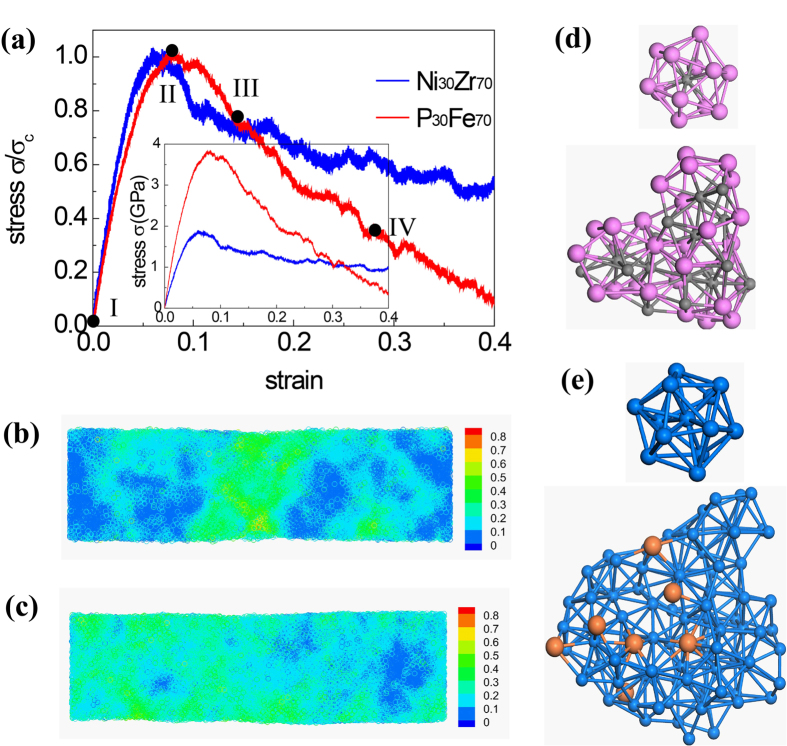
(**a**) Normalized stress-strain profiles of Ni_30_Zr_70_ and P_30_Fe_70_ under uniaxial stretching. Inset is the original stress-strain profiles. Points I to IV, in turn, correspond to the strain of 0, 0.068, 0.144, and 0.29. Equivalent strain contours for deformed P_30_Fe_70_ and Ni_30_Zr_70_ at state III are shown in (**b**) and (**c**), respectively. (**d**) One Ni-centered full icosahedral (FI) cluster and one NiZr supercluster consisting of 6 FI clusters. (**e**) One Fe-centered FI cluster and one PFe supercluster consisting of 19 FI clusters.

Two MG nanowire samples, Ni_30_Zr_70_ and P_30_Fe_70_, are studied respectively with MD simulations using LAMMPS^22^. Figure 1a shows the stress-strain profiles during the elongation for Ni_30_Zr_70_ and P_30_Fe_70_ MGs. For comparison the stresses are normalized by their respective yield strength *σ*_*c*_. It is seen that after yielding, the stress of P_30_Fe_70_ nanowires drops abruptly to one-tenth of *σ*_*c*_ as the strain increases to 0.4 (Fig. 1a, the red line), indicating brittle nature of the material. For Ni_30_Zr_70_, however, the stress drops mildly to about one half of *σ*_*c*_ as strain reaches to 0.4, indicating the material is relatively ductile. Fig. 1b,c show the close-up view of the two samples at *ε* = 0.144. The color of individual atoms represents the local von Mises equivalent strain^23^. The results show that during elongation, P_30_Fe_70_ glass develops one localized shear zone across the sample (Fig. 1b) while Ni_30_Zr_70_ glass has multiple small shear bands distributing along the sample. The size and the distribution of shear bands reveal distinct strain softening mechanism: for P_30_Fe_70_ the softening is mainly due to the localized deformation; for Ni_30_Zr_70_ however, softening is due to the initiation of multiple small bands which gives rise to rather homogeneous deformation in the sample.

We are interested in the mechanisms that regulate the initiation and propagation of tiny shear bands. One key question is why there emerge diffusive shear bands in ductile Ni_30_Zr_70_ rather than localized bands seen in P_30_Fe_70_ sample. One possible way to reveal the mechanism is to examine the internal structure of glasses during deformation at different stages. It has been shown the SRO and their medium-range connections, *i.e.*, the atomic full icosahedra (FI) formed superclusters, would directly affect the mechanical behavior of MGs^17^,^18^,^20^,^24^,^25^,^26^. Here we also adopt the scheme and use the degree of SRO as the indicator to probe the properties of MGs. Figure 1d,e show the examples of superclusters for NiZr and PeFe samples, where FIs interpenetrate each other. The capability of forming those FI superclusters serves as an indicator for plastic deformability, which will be illustrated in detail next.

**Figure 2:**
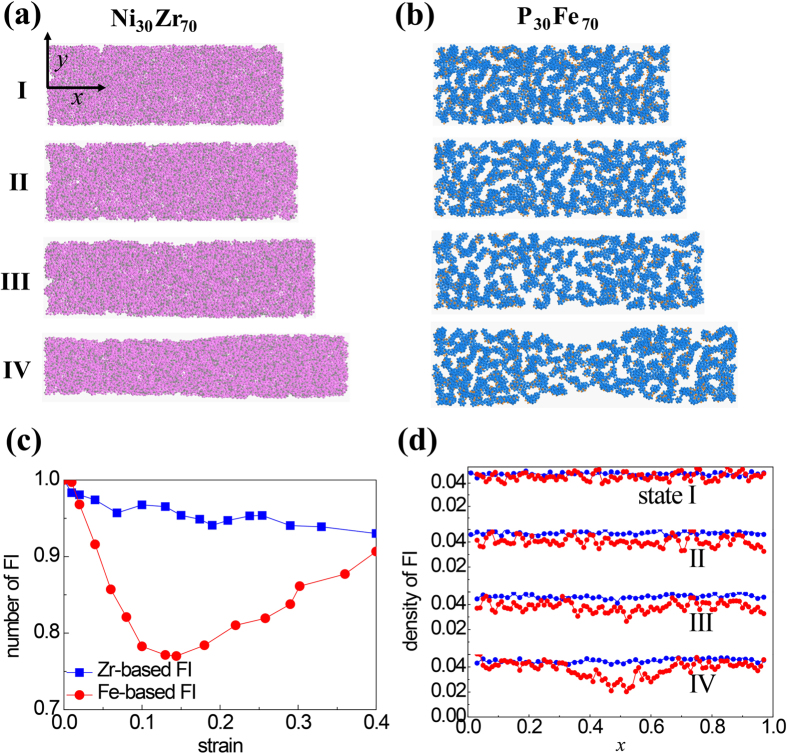
Snapshots of the networks of FI clusters under uniaxial tension for (**a**) Ni_30_Zr_70_ and (**b**) P_30_Fe_70_ glasses. For clarification only atoms in FI superclusters within a slab of 1 nm thick are presented. (**c**) Variation of normalized number of FI during the deformation of MG nanowires. (**d**) The distribution of FI along the axial direction at different strains. The blue dots represent the Zr-based MGs while the red one is for Fe-based MGs.

Figure 2a,b show some snapshot configurations of both glasses during stretching. For clarification only the atoms in FI superclusters within a slab of 1 nm thick are shown. The states I-IV are the deformation snapshots when ε = 0, 0.08, 0.144, 0.29, respectively. It is seen that at state I, *i.e. ε* = 0, Ni_30_Zr_70_ sample has relatively dense network of FI clusters compared to that in P_30_Fe_70_ sample. As the sample is stretched, the network in P_30_Fe_70_ becomes even sparse (state II, III, and IV), which is in distinct contrast to the situation in Ni_30_Zr_70_ where the network remains dense. It seems there exists a strong correlation between FI network and ductility: when the network becomes sparse due to the breakage of FI supercluster linkage, the glass shows brittle behavior in stretching.

To quantitatively analyze the stretch induced change for FI networks, we count the number of FI clusters during stretching. As seen in Fig. 2c in Ni_30_Zr_70_ the population of Zr-centered FI clusters decreases mildly (<7% when *ε* = 0.4). For P_30_Fe_70_, however, the population of Fe-based FI clusters first drops quickly then recovers during plastic yielding and eventually reaches to about 90% as *ε* = 0.4. We attribute the transition of FI cluster population to the propagation of STZ, which releases the stored strain energy and enables the reformation of FI clusters. Since the propagation of STZ is highly localized, we summarize the distribution of FI clusters along the axis of the nanowires in Fig. 2d to further capture the homogeneous/heterogeneous deformation behavior of FI network. It is seen that for Ni_30_Zr_70_, the density of FI clusters along the axis of nanowire remains almost constant at each state of I-IV. For P_30_Fe_70_, at states III and IV, the distribution of FI is highly differentiated, indicating the breaking of FI networks is localized and deformation becomes heterogeneous.

### Fracture behavior of MGs

**Figure 3:**
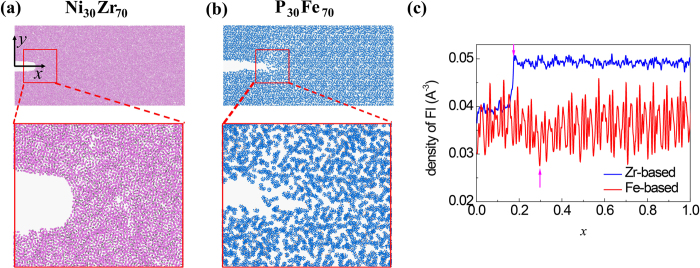
Snapshots of networks of FI clusters for (**a**) Ni_30_Zr_70_ BMG under nominal strain *ε* = 0.09 and (**b**) P_30_Fe_70_ BMG under loading strain *ε* = 0.05. For clarification only the atoms in FI superclusters are presented. (**c**) The distribution of FI along the axial direction at*ε* = 0.09 and *ε* = 0.05 for Ni_30_Zr_70_ and P_30_Fe_70_ glasses, respectively. The blue line represents the Zr-based MGs while red one for Fe-based MGs.

Since the tensile ductility is closely related to the fracture toughness of materials, we repeat the simulations described in the work by Murali *et al.*^21^ to check if the evolution of SRO structure would affect the fracture behavior of MGs. In the new simulation systems, P_30_Fe_70_ and Ni_30_Zr_70_ samples with dimensions of 200 nm × 100 nm × 2 nm are constructed by multiplying the quenched glass unit as obtained in the nanowire samples. The samples are loaded in tension along *y*-direction after introducing a sharp crack with initial length 34 nm. All the other details are the same as those described in Ref 21. From the results we also observed the distinct fracture behavior of Ni_30_Zr_70_ and P_30_Fe_70_ BMGs: the former is ductile while the latter is brittle, as described in Ref 18. With the MRO structure presented at specific strain (Fig. 3), it is seen at the crack tip that the Ni_30_Zr_70_ sample has dense network of FI clusters, which effectively prohibits the propagation of crack. For P_30_Fe_70_ sample, however, the network of FI clusters at the crack tip is sparser than those far from the tip, indicating the network of FI clusters at the tip is destroyed while crack propagating. Figure 3c shows the density of FI along the *x*-direction where one apparent drop occurs at the crack tip (pink arrow). For Ni_30_Zr_70_ sample, however, the FI density has no obvious change at the crack tip. We note that the drop ahead of the crack tip is due to the increased volume of the crack. So for bulk metallic glasses, the fracture behavior also has strong correlation with FI networks, which is similar to the case of nanowires under stretching.

### Reformation capability of SRO and their medium-range connections

**Figure 4:**
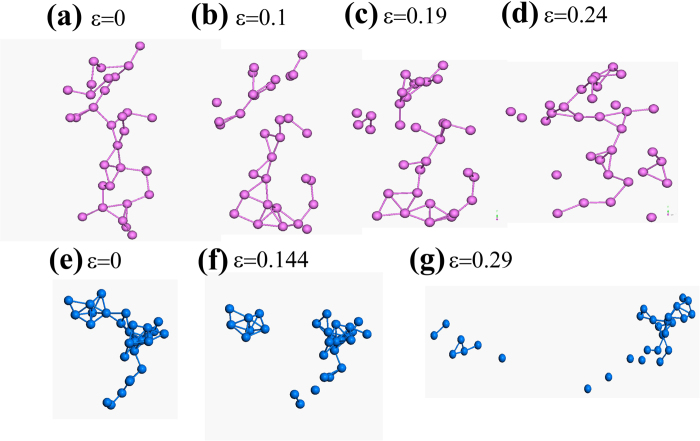
(**a–d**) Snapshots of one Zr-based FI supercluster within the STZ under uniaxial stretching. (**d–g**) Snapshots of one Fe-based FI supercluster within the STZ under uniaxial stretching. For clarity only the central atoms of FIs are shown here.

One question remaining elusive is what induces the distinct behavior of FI networks of the two BMGs. We have shown that the variation in population of FI clusters during stretching Ni_30_Zr_70_ nanowire is small (Fig. 2c). One may postulate that the network of Ni_30_Zr_70_ FI clusters could resist breakage due to their strong bonding. However, it seems to be contradictory to our observation that P_30_Fe_70_ has even higher resistance than that of Ni_30_Zr_70_ (Fig. 1a, the inset). Furthermore this postulation is also inconsistent with the observation by Lee *et al.*^20^ that the disruption of network would result in strain localization and lower plasticity. Here we propose that the reformation capability of FI networks has major role in regulating the ductility of glasses. We note that in the work of Shimizu *et al.*^27^, they have proposed there is a rejuvenation zone ahead of the crack tip, where the glass undergoes transition from well-aged state to rejuvenated state. To illustrate this mechanism, we examine the evolution of typical interpenetrating connections of FI networks within STZs for the two glasses, as shown in Fig. 4. For Ni_30_Zr_70_ glass, the FI network does break in the backbone during stretching (Fig. 4b). Yet the broken backbones form a long backbone again (Fig. 4c,d). Such reformation behavior, however, is not seen in P_30_Fe_70_ FI networks: as the strain increases, the broken backbone of the network is continuously falling into segments (Fig. 4f,g).

**Figure 5:**
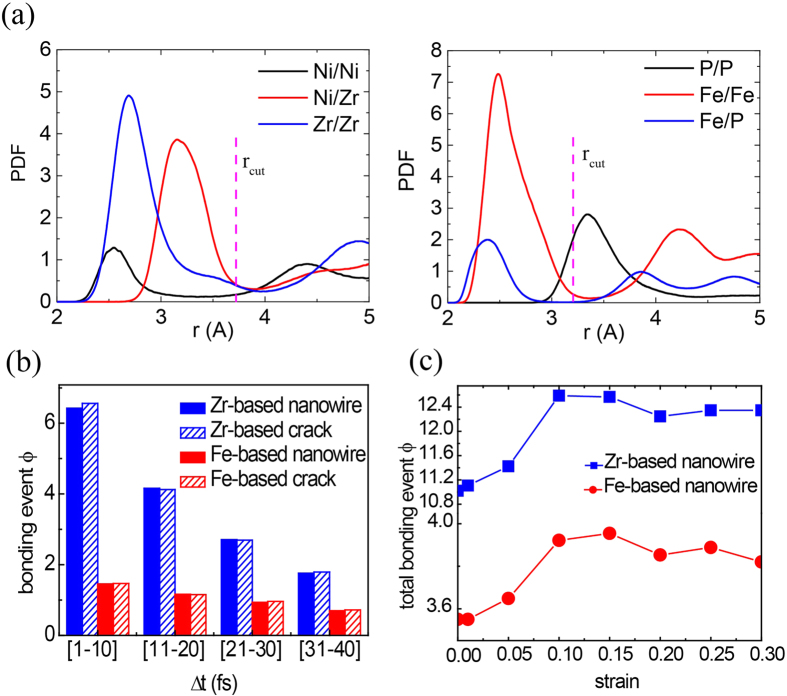
(**a**) Pair distribution functions for Ni_30_Zr_70_ (left) and Pe_30_Fe_70_ (right) glasses. The parameter, *r*_cr_, is the distance to the first valley in the PDF profile. (**b**) The bonding event between individual FIs for nanowires and bulk MGs containing crack at different time interval of 1–10 fs, 11–20 fs, 21–30 fs etc. The blue color indicates Zr-based MGs and the red one for Fe-based ones. The strain is *ε* = 0.144 for Zr- and Fe-based nanowries, *ε* = 0.09 and *ε* = 0.05 for Zr- and Fe-based MG cracks, respectively. (**c**) The evolution of total bonding event occurred within 1 ps during the stretch of nanowires.

To further distinguish the reformation capability of FI networks between Zr- and Fe-based glasses, we count the bonding event between two interpenetrating Fe-/Zr-centered FIs during loading of nanowires and bulk MGs (see Methods for details). Figure 5b shows the bonding event *ϕ* occurring at different time interval of 1–10 fs, 11–20 fs, 21–30 fs, etc in the stretched nanowires as *ε* = 0.144. It is seen that in Zr-based glass, the bonding events are much higher than those in Fe-based sample, indicating the Zr-based sample has much higher reformation capability. For a bulk MG with a crack, the bonding event has the same trend as that in nanowires (Fig. 5b). We further count the total bonding event occurred within 1 ps at various stretched state for nanowires (Fig. 5c). It is seen that the bonding event for Zr-based glasses keeps higher than that of Fe-based ones during the stretch. Such reformation capability is thought to promote reconnection of FI clusters and reformation of MRO networks, subsequently enhances the ductility of glasses. In the calculations, we have selected Fe-/Zr-centered full icosahedrons as the connection units since the population of such FIs is dominant: the fraction of Zr-centered FIs reaches as high as 89% and that of Fe-centered FIs reaches almost 100%. In Ni_30_Zr_70_ glasses, we also calculate the bonding propensity of Ni-centered FIs and the results show the difference for bonding propensity between Ni-centered and Zr-centered FIs is within 4%. Here we have considered the connection as the interpenetrating connection of icosahedra (ICOI)^20^. To further see how the other connection types, i.e. vertex sharing (VS), edge sharing (ES) and face sharing (FS)^26^, are affected in the calculating of bonding event, we add more calculations (see Methods). The results show the bonding event for non-ICOI has mild change: for P_30_Fe_70_ glass, the bonding event of ICOI is about 3.92 while that of non-ICOI is about 5.04; for Ni_30_Zr_70_ glass, the bonding event of ICOI is about 12.59 while that of non-ICOI is about 10.8. We also change the time interval from 1 fs to 10 fs and keep the total time as 1 ps (see Methods), the results show that the bonding event decreases accordingly: it changes from 12.59 to 9.2 for Ni_30_Zr_70_ glass, and from 3.92 to 3.6 for P_30_Fe_70_ glass. It is seen that the P_30_Fe_70_ glass still has much lower bonding event.

### Cooling rate/free volume effect on the rejuvenation capability

**Figure 6:**
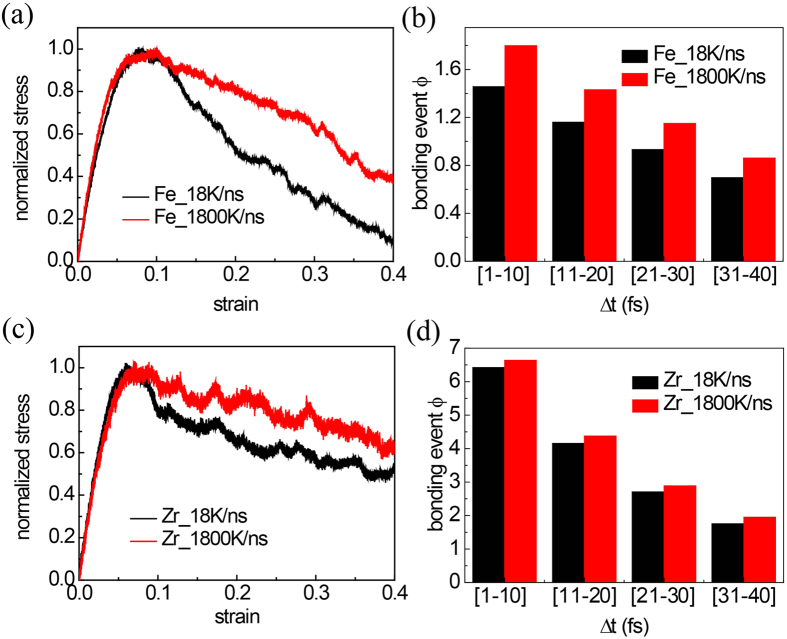
(**a**) Normalized stress-strain profile of P_30_Fe_70_ samples prepared at different cooling rate, and (**b**) the bonding event between individual FIs at different time interval of 1–10 fs, 11–20 fs, 21–30 fs etc. (**c**) Normalized stress-strain profile of Ni_30_Zr_70_ samples prepared at different cooling rate, and (**d**) the bonding event between individual FIs at different time interval of 1–10 fs, 11–20 fs, 21–30 fs etc.

To further investigate if such high reformation capability is possessed solely by ductile glasses, we change the simulation protocol with additional simulations for P_30_Fe_70_ nanowires. The new sample prepared at cooling rate of 1800 K/ns which is two orders of magnitude higher than the previous ones. The results show that the ductility for the fast-cooling-rate sample is higher than that of the slow-cooling-rate one (Fig. 6a). Since fast cooling rate would lead to more free volume or higher concentration of vacancies, our results are consistent with previous observation that pre-existing shear bands/defects can enhance the ductility of BMGs^10^,^13^. Through comparing the evolution of FI clusters, we find the FI bonding propensities are higher in fast-cooling-rate Fe-based MGs (Fig. 6b). We also repeat this protocol on Ni_30_Zr_70_ glass and find the same trend that fast-cooling rate enhances the bonding propensity (Fig. 6c,d). Thus the reformation capability for the fast-cooling samples is higher than that in the slow-cooling ones. It seems that the reformation capability depends not only on the components, but also on free volume of glasses. The former determines if the glasses would form dense FI clusters (like Zr-based glasses), which enhances the possibility for isolated FIs to connect each other. The latter, however, determines if the FIs could overcome steric hindrance to establish connection.

### Temperature effect on the reformation capability

**Figure 7:**
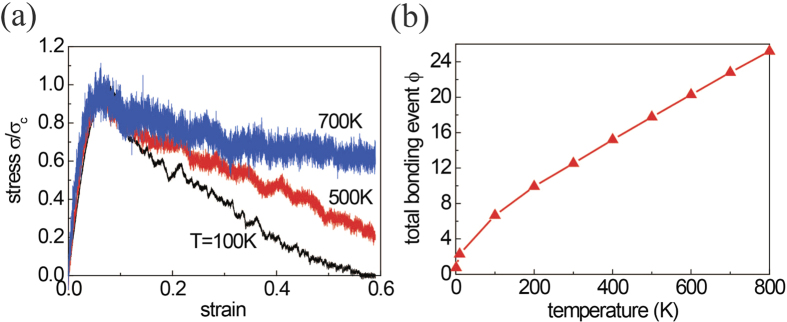
(**a**) Normalized stress-strain profile of Ni_30_Zr_70_ nanowire stretched at different temperatures. (**b**) The total bonding event between individual FIs for glasses stretched at different temperatures as *ε* = 0.144.

Apart from the cooling rate/free volume effect, the temperature during loading is another factor influencing the deformability of MGs. It is well known that the increase of testing temperature results in the brittle-to-ductile transition of MGs^14^. To see if such temperature-induced transition has correlation with the bonding propensity of FIs, we repeat the simulations of stretching Ni_30_Zr_70_ nanowires at various temperatures. From the stress-strain profiles (Fig. 7a), it is seen that the ductility of MGs enhances as temperature increases. Meanwhile, the corresponding bonding event *ϕ* of FIs also increases with the temperature (Fig. 7b), indicating the temperature could enhance the bonding propensity efficiently. Subsequently, the re-bonding of FIs greatly enhances the ductility of MGs. Such observed temperature dependent bonding propensity can be explained by Arrhenius theory, 
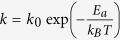
, where *k* is the bonding reaction rate, *E*_*a*_ is the activation energy, *k*_*B*_ is the Boltzmann constant, and *T* the temperature. It proves again that the deformability has strong correlation with the reformation capability of SRO structures.

## Discussion

In the comparative study we have used FI, *i.e.* a cluster of atoms with coordination number 12 (CN = 12), as the index to monitor the propensity of reformation of glasses. Although for Zr-based glasses the population of FIs is demonstrated to be dominant^17^,^18^, there is still a lack of such information for Fe-based glasses. To elucidate it we calculate the number of clusters with different CN for P_30_Fe_70_ and find the fraction of FIs (CN = 12) in all clusters is about 52%, much higher than that of the second highest cluster with CN = 11 (19.6%). So it is appropriate to use FI as the index in calculating the propensity of reformations of glasses.

To extend the finding that ductility of Fe- and Zr-based MGs is induced by the reformation capability of MRO structures, we also conduct some additional calculations based on published result. It has been shown that the strength and ductility of Cu_*x*_Zr_100−*x*_ MGs vary with the Cu composition: the strength increases while ductility decreases with *x*^28^. We calculate the bonding event *ϕ* for different *x* (*x* = 20, 30, 46, 64) during the deformation. The results agree with our conclusion that high bonding event *ϕ* leads to high ductility (see Supplementary Material). So for some typical studied MG samples, we find there is strong correlation between ductility and reformation capability of SRO structures. With this hint, we may explore further to see if such correlation is general to all the MG glasses.

We note that in the work by Murali *et al.*^21^, the fluctuation in the stress field near a crack tip due to atomistic level structural inhomogeneity was believed to govern the brittle fracture behavior. Our observations could potentially explain why there is so much fluctuation in strength of cavitations in brittle BMG. With MRO becoming more sensitive to stretch, the strength of cavitations of material would presumably become more sensitive to the local atomic environment: with confinement of dense FI clusters, the fluctuation in strength of cavitations is effectively alleviated.

In summary, we have studied the ductile versus brittle mechanisms of Zr- and Fe-based metallic glasses through molecular dynamics simulations. With the degree of SROs and their medium-range connections as an indicator, we have shown that the brittle behavior of Fe-based glass is associated with the dramatically decrease of FI clusters, which induces the initiation and propagation of STZs. For Zr-based glass, however, our results show that the degree of FI clusters has mild change during the stretch. We propose that, instead of the strength of networks of FIs, the reformation capability to form networks of FIs plays the major role in regulating the ductility of glasses. For glasses with high reformation capability, the FIs can re-bonding instantaneously after the breakage of FI networks, which effectively prevents the propagation of STZs and the heterogeneous deformation of glasses. Our study provides important insights into the understanding about the mechanisms accounting for ductility or brittleness of BMGs.

## Methods

### MD simulations

Atomic interactions are modeled by embedded atom method (EAM) potentials with parameters given by Mendelev *et al.*^29^,^30^. Glass samples consisting of 16000 atoms with randomly substituted solid solution in a face-centered cubic (FCC) lattice are used in a melting-and-quenching simulation, whose temperature is raised gradually from 0 to 2100 K and is then cooled down to 300 K. The cooling rate is set at 18 K/ns and the periodic boundary condition (PBC) is applied in all directions. The samples are then replicated three times along *x*-direction to form nanowires following 1 ns equilibrium at 300 K with the PBC applied along *x*-direction only. The time step for integration is chosen to be 1 fs. In all simulations the lateral pressure is controlled at 0 bar. The MGs nanowire samples with final dimensions of 20 nm × 6.7 nm × 6.7 nm are then loaded under uniaxial tension at the strain rate of 10^8^ s^−1^.

### Bonding event calculations

A criterion to judge the occurrence of bonding is defined that when two isolated FIs approach each other within a critical distance, *r*_cr_. To determine *r*_cr_, we calculate the pair distribution function (PDF) of Fe- and Zr-based MGs as shown in Fig. 5a. For the case of interpenetrating connection of icosahedra (ICOI), *i.e.*, a Fe-/Zr-centered FI overlapping with another Fe-/Zr-centered FI, *r*_cr_ is the distance to the first valley in the PDF profile (Fig. 5a). Via this approach, we obtain *r*_*cr*_ = 3.2 Å and *r*_*cr*_ = 3.7 Å for Fe- and Zr-based MGs, respectively. For other non-ICOI like vertex sharing (VS), edge sharing (ES) and face sharing (FS), the *r*_cr_ is doubled. To analyze bonding events, in each of the MD simulations we track all atoms’ trajectory at each femtosecond (fs) for one picosecond (ps). At time *t*_*i*_ +Δ*t* (*i* = 1,…1000), the structure deforms from its reference configuration (at time *t*_*i*_) and the bonding events are counted with the criterion. Here we define a parameter, *ϕ*_*i*_(*t*_*i*_, Δ*t*), to indicate the propensity of bonding, which is the bonding event occurred in 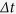 normalized by the mean number of FIs in the glasses. Then 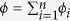 counts the total bonding events occurred within time interval Δ*t*.

## Additional Information

**How to cite this article**: Li, C. *et al.* Reformation Capability of Short-Range Order and Their Medium-Range Connections Regulates Deformability of Bulk Metallic Glasses. *Sci. Rep.* **5**, 12177; doi: 10.1038/srep12177 (2015).

## Supplementary Material

Supplementary Information
