# Supplementary material for: Reformation Capability of Short-Range Order and Their Medium-Range Connections Regulates Deformability of Bulk Metallic Glasses
Source: Sci Rep. 2015 Jul 16;5:12177. doi: 10.1038/srep12177 (PMC4648402; doi:10.1038/srep12177)
Supplement: Supplementary Information [file srep12177-s1.pdf]

# Supplementary Material for

# Reformation Capability of Short-Range Order and Their Medium-Range Connections Regulates Deformability of Bulk Metallic Glasses

Congling Li[1,2], Yujie Wei[1], Xinghua Shi[1*]

[1]LNM, Institute of Mechanics, Chinese Academy of Sciences, Beijing 100190
[2]School of Civil Engineering, Luoyang Institute of Science and Technology, Luoyang, Henan Province 471023

*shixh@imech.ac.cn

It has been shown that the strength and ductility of $Cu_xZr_{100-x}$ MGs vary with the Cu composition: the strength increases while ductility decreases with $x$ [s1]. We calculate the bonding event $\phi$ of FIs for different $x$ ($x$=20, 30, 46, 64) during the deformations. The calculating protocol is the same as described in the main text. It is seen that the bonding event decreases with $x$ (SFig. 1a), indicating the ductility has strong correlation with the bonding event of FIs, which is consistent with the finding for Fe- and Zr-based MGs. Meanwhile, the cooling rate also has influence on the rejuvenation capability, with fast-cooling-rate induces higher bonding event, in consistent with the finding for Fe- and Zr-based MGs.

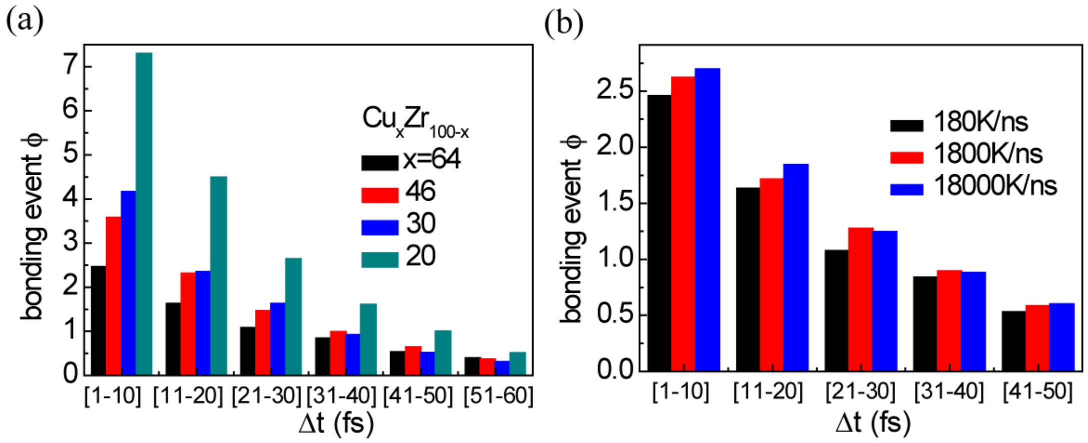


SFigure 1: (a) The bonding event between individual FIs at different time interval for $Cu_xZr_{100-x}$ nanowire. (b) The bonding event for $Cu_{64}Zr_{36}$ with different cooling rates.

[S1] Y. Q. Cheng, A. J. Cao, H. W. Sheng, E. Ma, Local order influences initiation of plastic flow in metallic glass: Effects of alloy composition and sample cooling history. Acta. Mater. 56, 5263 (2008).
